# Evaluating Person-Centred Integrated Care to People with Complex Chronic Conditions: Early Implementation Results of the ProPCC Programme

**DOI:** 10.5334/ijic.7585

**Published:** 2023-12-13

**Authors:** Miquel À. Mas, Ramón Miralles, Maria J. Ulldemolins, Ria Garcia, Sonia Gràcia, Josep M. Picaza, Mercedes Navarro Fernández, Maria A. Rocabayera, Montserrat Rivera, Núria Relaño, Mireia Torres Asensio, Pilar Laporta, Celia Morcillo, Laura Nadal, Ramona Hervás, Dolors Fuguet, Cristina Alba, Núria Miralles Banqué, Sònia Jimenez, Miriam Moreno Moreno, Carmen Nogueras, Helena Manjón Navarro, Rosa López, Guillem Hernández, Francesc López-Seguí, Laura Ricou Ríos, Arnau Pons, Nuria Prat, Jordi Ara Del Rey, Oriol Estrada

**Affiliations:** 1 Direcció Clínica Territorial de Cronicitat Metropolitana Nord, Institut Català de la Salut, Catalonia, Spain; 2 Department of Geriatrics, Hospital Universitari Germans Trias i Pujol, Badalona, Catalonia, Spain; 3 Direcció d’Atenció Primària Metropolitana Nord, Institut Català de la Salut, Catalonia, Spain; 4 Servei d’Atenció Primària Barcelonès Nord, Institut Català de la Salut, Catalonia, Spain; 5 Equip d’Atenció Primària Sant Roc i Equip d’Atenció Primària Gorg, Badalona, Catalonia, Spain; 6 Equip PADES Badalona, Institut Català de la Salut, Badalona, Catalonia, Spain; 7 Equip d’Atenció Primària Sant Adriàde Besòs, Sant Adriàde Besòs, Catalonia, Spain; 8 Equip d’Atenció Primària Llefià, Badalona, Catalonia, Spain; 9 Equip d’Atenció Primària Bufalà, Badalona, Catalonia, Spain; 10 Equip d’Atenció Primària Badalona Centre-Dalt de la Vila, Badalona, Catalonia, Spain; 11 Equip d’Atenció Primària El Masnou, El Masnou, Catalonia, Spain; 12 Equip d’Atenció Primària Ocata, El Masnou, Catalonia, Spain; 13 Unitat de Treball Social, Hospital Universitari Germans Trias i Pujol, Badalona, Catalonia, Spain; 14 Unitat d’Hospitalitzacióa Domicili, Hospital Universitari Germans Trias i Pujol, Badalona, Catalonia, Spain; 15 Direcció d’Organitzaciói Sistemes d’Informació, Gerència Territorial Metropolitana Nord, Institut Català de la Salut, Catalonia, Spain; 16 Gerència Territorial Metropolitana Nord, Institut Català de la Salut, Catalonia, Spain; 17 Research Group on Innovation, Health Economics and Digital Transformation (IGTP), Barcelona, Spain; 18 Centre de Recerca en Economia de la Salut, Barcelona, Spain; 19 eXiT Research group –trol Engineering and Intelligent Systems (IIiA –UdG), Girona, Spain

**Keywords:** patient-centred care, integrated delivery of health care, frailty, health resources, Health Impact Assessment

## Abstract

**Introduction:**

The evaluation of integrated care programmes for *high-need high-cost* older people is a challenge. We aim to share the early implementation results of the ProPCC programme in the North-Barcelona metropolitan area, in Catalonia, Spain.

**Methods:**

We analysed the intervention with retrospective data from May 2018 to December 2021 by describing the cohort complexity and by showing its 6-months pre-post impact on time spent at home and resources used: primary care visits, emergency department visits, hospital admissions and hospital stay.

**Findings:**

264 cases were included (91% at home; 9% in nursing homes). 6-month pre vs. 6-months post results were (mean, p-value): primary care visits 8.2 vs. 11.5 (p < 0.05); emergency department visits 1.4 vs. 0.9 (p < 0.05); hospital admissions 0.7 vs. 0.5 (p < 0.05); hospital stay 12.8 vs. 7.9 days (p < 0.05). Time spent at home was 169.2 vs.174.2 days (p < 0.05).

**Conclusion:**

Early implementation of the ProPCC programme results in an increase in time spent at home (up to 3%) and significant reductions in emergency department attendance (–37.2%) and hospital stays (–38.3%). The increased use of primary care resources is compensated by the hospital resources savings, with a result in the average total cost of –46.3%.

## Introduction

In the last decades, several initiatives at the national and regional levels have been developed to improve the care provided to people living with complex and advanced chronic conditions and high levels of health and social complex needs [1, 2]. Despite initial positive results of integrated care programmes tailored to high-need high-cost populations, in terms of improvement of quality care and cost-effective outcome, innovations on quality improvement initiatives are heterogeneous and the implementation of new models in real life is complex [3]. Potential person-centred benefits are difficult to rise due to barriers at clinical, operational, and financial challenges that different teams and institutions face during the process of engagement to new care models [4].

Several approaches have been used to assess the impact of innovative integrated care programmes, not only in terms of reducing the use of resources, but also in terms of person centricity. In relation to this, in the last years, different authors have studied time spent at home as the primary indicator of the impact of community-based integrated care [5], especially for the population in their last stage of life [6,7].

In Catalonia, the Metropolitana Nord Chronic Care Management Team at Institut Català de la Salut (Catalan Health Institute), the main health public provider in our region, has developed a strategy to improve the quality of care to chronic complex patients and patients with advanced illness. They developed an innovative care model that emerged from the Community Based Integrated Care Programme for People with Complex Chronic Conditions (ProPCC Programme) [8,9]. The theoretical frame of our project can be found in a previous publication in this journal [10]. In this context, we analysed the ProPCC by describing the complexity profile of an early implementation cohort, and by showing its impact on time spent at home and resources used.

## Description of the Care Practice

### Development of the Case Management Programme

The project started in 2018 with the elaboration of an integrated care clinical programme (the ProPCC Programme). The process designed included a qualitative study to explore patients’ and caregivers’ experiences and views, and a task group with professionals to validate evidence-based key actions to adapt them to their local contexts. A recent paper published in this journal [10] presents the framework of the integrated care model and the key characteristics of the programme, including 63 clinical actions to respond to the patients, caregivers, and professionals’ needs.

### Key Points for the Implementation Process

Through 2018, several organisational changes were defined in our institutions towards the achievement of this new model. The clinical actions included in the ProPCC Programme were used as a tool to guide the implementation, held in 16 of 64 primary care teams from our urban territory in the North-Barcelona metropolitan area in Catalonia (Spain). The implementation was focused not only on the Catalan Health Institute units, at primary care and hospital settings (vertical integration), but also on other territorial services, such as local municipality services, community-based teams and community-based rehabilitation services (horizontal integration).

Firstly, new Case Management Multidisciplinary Teams (CM-MDT), with social and health care staff experts, emerged from primary care (Primary Care Service Barcelonès Nord) and hospital care (Hospital Universitari Germans Trias i Pujol) from the Catalan Health Institute. In primary care centres, every single primary care team created a CM-MDT unit formed by a physician, a nurse, and a social worker. The Geriatric Department of the Hospital Universitari Germans Trias i Pujol created a CM-MDT formed by a geriatrician, a geriatric nurse, and a social worker, to guarantee support to primary care teams in the management of crises and transitions. This team was responsible for case management organisation during hospital care: starting with the decisions taken during the admission phase in the emergency care, as well as implementing any actions that were urged from the clinical programme. This was done in collaboration with reference primary care professionals and clinicians from other specialties, with a proactive approach. Primary Care CM-MDT unit leadership pivoted on nurses who provided education on care and transitional support. PC CM-MDT physicians were mainly responsible for health crisis treatments, in collaboration with other members of the primary care network, hospital-at-home teams, day hospitals and other outpatient services of the reference acute and intermediate care hospitals. Moreover, they had a key role in the identification of advanced illness stages and the definition of person-centred care based on the person’s preferences and viewpoints. PC CM-MDT social workers were responsible for the communication with members of social services from the municipalities and community services, in order to individualise care plans and activate social resources based on people’s needs and views.

Secondly, multidisciplinary local meetings in the format of case conferences (CC-ProPCC) were implemented to support professionals and teams in the elaboration of person-centred care plans and in the decision-making process for high complexity cases. PC CM-MDT organised weekly case conferences (CC-ProPCC 1) at primary care centres, focused on the identification of individuals with high needs (frail patients, complex chronic patients, and patients with advanced illness). Person-centred case plans were tailored, following the strategic recommendations of the Catalan Ministry of Health [11]. Every two weeks, PC CM-MDT held a case conference (CC-ProPCC 2) at primary care centres oriented to horizontal integration, with the participation of physicians, nurses, social workers of the team and other teams such as hospital-at-home, palliative care at home units and home rehabilitation services. These meetings were open to social services members and other participants involved in community care. Hospital-based CM-MDT handled the communication with other specialty teams when cases required support from hospital-based teams and had a key role in the development of case conferences oriented to vertical integration. Biweekly, other meetings were celebrated in reference hospitals (CC-ProPCC 3), to support decision making in selected cases with high complexity.

Thirdly, an intense collaboration between PC and hospital-based CM-MDT, with reference teams from several specialities, and community services, were urged in cases of advanced illness; in order to ease maintaining patients at home at end-of-life by coordinating response to crises and by minimising time at the hospital, when possible, in cases with clear progression of the clinical trajectory to end-of-life stages. One of the examples of this collaboration was the integration of care between primary care CM-MDT, hospital-based CM-MDT, hospital-at-home units and home-based palliative care teams for people at end-of-life.

## Evaluation Methodology

The impact of introducing the ProPCC programme has mainly been evaluated on the time spent at home and on the use of healthcare resources in the population where it has been implemented [9]. A retrospective observational analysis has been carried out with information obtained from the administrative database of the care devices in the region from May 2018 to December 2021. It should be noted that during the response to the health emergency of the first wave of COVID-19, the inclusion of new cases dropped (without stopping completely). This study has been approved by the Medicines Research Ethics Committee IDIAP Jordi Gol, with code number 22/084-P.

An initial descriptive analysis of the population characteristics has been carried out considering age, gender, main diagnoses, Complex Chronic Patients (CCP) or patients with Advanced Chronic Disease (ACD) complexity profiles, adjusted morbidity groups (GMA) [12] and usual residence (own home or nursing home). The healthcare resources on which the impact has been analysed from, are primary care visits (physician, nurse, social worker and continuing and urgent care), hospital care use (emergency department visits, hospital admissions and hospital stay) and days spent at home.

On the one hand, to evaluate the effectiveness of the programme, the differences in the means of the previously mentioned variables were estimated in the 6 months before and after the inclusion of a patient in the programme. A paired sample t-student before-after was used to calculate the differences, considering them statistically significant if their p-value was less than 0.05.

On the other hand, it has been estimated the impact that the programme has had on the costs of the different healthcare services (primary and hospital) measured in monetary units (euro, year 2020). The unit prices of each healthcare resource have been taken from the public prices of the Catalan Health Service [13]. A discount rate was not used due to the limited follow-up period. In relation to the average costs per patient of visits to the physician, nurse, social worker, continuous and urgent care and hospital care, they have been calculated by multiplying the unit price of each benefit (€50, €35, €30, €105 and €215, respectively) by the average number of times a patient uses it. The average cost per patient of hospitalisation has been calculated by multiplying the number of days an admission lasts on average by the average number of times a patient has been admitted by the daily price of a standard hospital stay (€751 during the first 5 days and €597 from the sixth).

## Results

### Population Study Characteristics

The 264 cases showed an average age of 83.6, 50.4% being women; the majority of patients (83%) correspond to the highest adjusted morbidity groups, and most were treated at home (9.1% lived in a nursing home). Table 1 shows the clinical characteristics of the population at the time of inclusion in the programme. Most patients presented an advanced chronic disease (55.7%) and the main inclusion criteria was presenting multimorbidity with a high risk of readmission (54.9%), difficulties in managing functional dependence (20.1%) and difficulties in managing an advanced disease process (19.3%). High mortality rate during the 6 months follow-up phase was present due to high prevalence of patients with advanced conditions at end-of-life: six months after inclusion in the programme one of every four patients passed away (with 3.78% mortality at 1-month follow-up and 18.22% between 1 and 6 months follow-up).

**Table 1:** Clinical characteristics of the implementation cohort (N = 264).


CLINICAL CHARACTERISTICS	N (%)

**Main clinical diagnoses grouped**	

Arterial hypertension	158 (59.8)

Skeletal muscle	156 (59.1)

Cardiological	112 (42.4)

Respiratory	92 (34.8)

Cognitive disorder/dementia	86 (32.6)

Diabetes Mellitus 2	83 (31.4)

Chronic kidney disease	75 (28.4)

**Complexity profile**	

Advanced chronic disease patients	147 (55.7)

Complex chronic patients	117 (44.3)

ProPCC Programme inclusion criteria*	

Multimorbidity with a high risk of readmission	145 (54.9)

Complex functional dependency	53 (20.1)

Advanced complex disease	51 (19.3)

Coping difficulties with polipharmacy	48 (18.2)

Frequent visits to the Emergency Department for the same reason	32 (12.1)

Difficulties accepting the lack of health	31 (11.7)

Caregiver burden	31 (11.7)

Coping difficulties in the care of patients living with dementia	23 (8.7)

**Adjusted morbidity groups (GMA)**	

Group 4	156 (83)

Group 3	30 (16)

Groups 1–2	2 (1)

**Place of residence**	

Own home (with family or external caregiver support)	240 (90.9)

Nursing home	24 (9.1)


* Patients could have more than one inclusion criterion.

### Clinical Activity Linked to the Programme

Table 2 shows the main changes in the healthcare model, based on case conferences, in which case management teams share care plans with the different community services that participate in the programme, and on home care resources activation for complexity management when needed.

**Table 2:** Multidisciplinary collaboration derived from the implementation of the programme (N = 264).


MULTIDISCIPLINARY COLLABORATION	N (%)

**Presentation of cases in multidisciplinary meetings**	

Case conference ProPCC 1 (primary care teams)	174 (66%)

Case conference ProPCC 2 (community based teams)	235 (89%)

Consultories ProPCC 3 (primary care and hospital-based teams)	35 (13.3%)

**Activation of community services**	

Palliative care at home	69 (26.1%)

Rehabilitation at home	47 (17.8%)

Hospital-at-home	22 (8.3%)

**Activation of hospital resources**	

Hospital-based Case Management unit	89 (33.7%)


### Impact on Time Spent at Home

The inclusion of patients in the programme was associated to a significant increase (of approximately 5 days) in the time spent at home in the six months after the intervention, with a 3% increase in time. This difference was slightly higher for surviving patients than for non-surviving patients (as shown in Table 3).

**Table 3:** Time spent at home 6 months before and after the inclusion in the programme, for the general population and according to survival.


	N (%)	DAYS AT HOME 6 MONTHS PRE-INTERVENTION*	DAYS AT HOME 6 MONTHS POST-INTERVENTION*	VARIATION (DAYS, %)	P VALUE

**Survivors**	206 (78%)	171.56 (17.28)	177.01 (11.31)	5.45 (3.17%)	**p < 0.05**

**Non survivors**	58 (22%)	167.90 (23.68)	172.66 (16.09)	4.76 (2.83%)	**p < 0.05**

**Total**	264 (100%)	169.23 (21.61)	174.24 (14.67)	5.01 (2.96%)	**p < 0.05**


* Variables are mean (Standard Deviation).

### Impact on the Use of Primary and Hospital Care Resources

Healthcare resource consumption in the sample was high: in the 12 months prior to inclusion in the programme, the cohort had 182 emergency department visits (68.93%) and 130 (49.24%) hospital admissions. This percentage was reduced during the 6 months after entering the programme, with 114 (43.18%) emergency department visits and 89 (33.71%) hospital admissions.

Table 4 shows the variations that have occurred in the use of healthcare resources (visits to primary care, emergency consultations, hospital admissions and their duration) as a result of including patients in the programme, comparing the six months before and after the start of the intervention, for the whole sample and segmented by survival.

**Table 4:** Variations in the use of healthcare resources 6 months before and after the inclusion in the programme, for the whole sample and after a segment-by-segment analysis based on survival.


WHOLE SAMPLE (N = 264)	RESOURCE	6-MONTHS PRE-INTERVENTION*	6-MONTHS POST-INTERVENTION*	MEAN VARIATION	P VALUE

**Primary care visits**	Physician	8.22 (8.28)	11.45 (13.39)	39.88%	**<0.05**

	Nurse	9.33 (11.90)	19.85 (19.34)	111.84%	**<0.05**

	Social worker	0.99 (2.74)	2.06 (3.65)	107.76%	**<0.05**

	Continuous/urgent care	0.71 (3.67)	0.72 (2.98)	1.12%	0.97

**Hospital care use**	Emergency department visits	1.39 (1.69)	0.87 (1.42)	–37.23%	**<0.05**

	Hospital admissions	0.70 (1.05)	0.49 (0.83)	–29.24%	**<0.05**

**Hospital Stay**	Hospital days	12.77 (21.63)	7.85 (14.75)	–38.29%	**<0.05**

**SURVIVORS (N = 206)**	**RESOURCE**	6-MONTHS PRE-INTERVENTION*	6-MONTHS POST-INTERVENTION*	**MEAN VARIATION**	**P VALUE**

Primary care visits*	Physician	7.77 (8.65)	12.19 (14.1)	56.89%	**<0.05**

	Nurse	8.54 (12.03)	21.25 (20.11)	148.83%	**<0.05**

	Social worker	0.65 (1.77)	2.14 (3.57)	229%	**<0.05**

	Continuous/urgent care	0.65 (4)	0.68 (3.3)	3.75%	0.97

**Hospital care use**	Emergency department visits	1.29 (1.6)	0.79 (1.39)	–38.76%	**<0.05**

	Hospital admissions	0.62 (0.96)	0.38 (0.75)	–39.3%	**<0.05**

**Hospital Stay**	Hospital days	10.85 (19.84)	6.47 (13.94)	–40.39%	**<0.05**

**NON-SURVIVORS (N = 58)**	**RESOURCE**	6-MONTHS PRE-INTERVENTION*	6-MONTHS POST-INTERVENTION*	**MEAN VARIATION**	**P VALUE**

Primary care visits*	Physician	9.81 (6.64)	8.85 (10.22)	–9.79%	**0.54**

	Nurse	12.16 (11.12)	14.91 (15.49)	22.62%	**0.27**

	Social worker	2.21 (4.63)	1.79 (3.97)	–19%	**0.61**

	Continuous/urgent care	0.88 (1.34)	0.87 (1.34)	1.14%	**1**

**Hospital care use**	Emergency department visits	1.78 (1.96)	1.17 (1.52)	–34.27%	**0.06**

	Hospital admissions	0.97 (1.3)	0.91 (0.98)	–6.19%	**0.81**

**Hospital Stay**	Hospital days	19.59 (26.1)	12.79 (16.56)	–34.71%	**0.09**


* Variables are mean (standard deviation).

The results show an important polarised resource variation. On the one hand, with the inclusion of patients in the programme, visits to primary care have increased significantly: they have done so in a statistically significant way in the disciplines of physician, with an increase of almost 40%, and nursing and social work, doubling in both cases. Visits to continuing and urgent care do not show a statistically significant variation. On the other hand, hospital care was reduced both in the use of emergency department and hospitalizations. Emergency department visits decreased by almost 40% and the number of admissions was reduced by almost 30%. This reduction in the number of hospital admissions was also accompanied by a 38% reduction in hospital days. All reductions are statistically significant. Therefore, results show a clear shift of resources from hospital care to primary care.

The segment-by-segment analysis performed based on survival, showed that the positive impact on hospital care resource use (reduction) is statistically significant in the pre-post outcomes in the survivors’ subgroup, but these positive results do not remain significant in the non-survivors’ sample.

### Impact on the Costs of the Healthcare Resources Used

**Figure 1:**
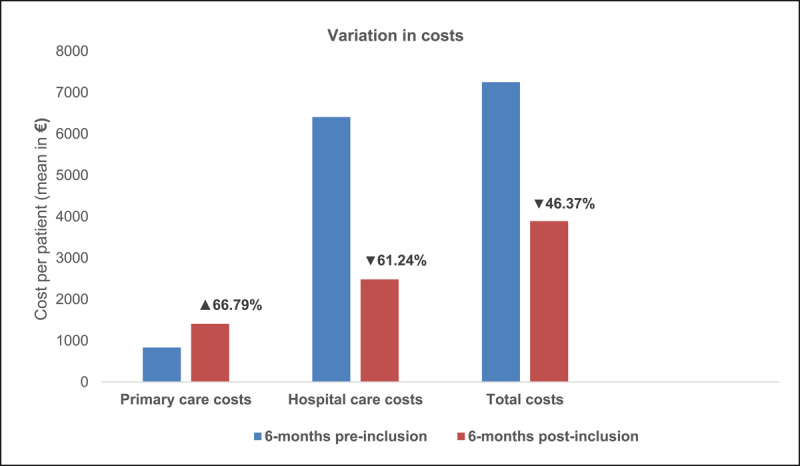
Variation in healthcare resources costs six months before and after inclusion in the programme, per patient.

Table 5 shows the variation in costs of healthcare resources use 6 months before and after the inclusion in the programme. During the six months prior to the implementation of the programme, each patient represented an average cost of €7,249. Hospitalisation was the biggest part of the total cost (84%). During the following six months after inclusion, each case represented an average cost of €3,889/patient (cost reduction of 46.37%). In this case, the increase of €563 in primary care resources per patient was compensated by the decrease of €3,923 in hospital care per patient. Thus, the weight that represents the cost of hospitalisation, although it remained the most important expense, it was reduced by 61.24%, at the expense of increasing primary care costs by 66.79% (see Figure 1).

**Table 5:** Costs related to healthcare resources six months before and after inclusion in the programme, per patient.


HEALTHCARE RESOURCES		6-MONTHS PRE-INTERVENTION*		6-MONTHS POST-INTERVENTION*		VARIATION*	
		
		€	% ON TOTAL	€	% ON TOTAL	€	% ON TOTAL

Primary care	Physician	411	5.67	573	14.72	162	39.42

	Nurse	327	4.51	695	17.87	368	112.54

	Social worker	30	0.41	62	1.59	32	106.67

	Continuous/urgent care	75	1.03	76	1.94	1	1.33

**Primary care total costs**		**843€**	**11.63**	**1,406€**	**36.16**	**563€**	**66.79**

Hospital care	Emergency department	299	4.12	187	4.81	–112	–37.46

	Hospital stay	6,107	84.26	2,296	59.06	–3,811	–62.40

**Hospital care total costs**		**6,406**	**88.37**	**2,483**	**63.86**	**–3,923**	**–61.24**

**Total costs per patient**		**7,249**		**3,889**		**–3,360**	**–46.37**


* Variables are mean and %.

## Discussion

The results derived from the analysis of the early implementation of our programme, show its impact on the increase in time spent at home thanks to the multidisciplinary case management model aimed at people with high needs. It is important to note that the programme implementation has not been done with an increase in specific resources but by resizing the resources used in primary care and hospital care (vertical integration) and intensifying collaboration with community services (horizontal integration). We see how the implementation shows a variation in the use of care resources. On the one hand, there is an increase in primary care visits, especially those of nursing and health social work. On the other hand, there is a reduction in hospital visits (emergency department and conventional hospitalisation) which may be related to the continuity of the case management process in the community and throughout the hospitalisation trajectory. As a result, the days of stay in the hospital are reduced while the days staying at home are increased. With this variation in the use of resources, because hospital resources are more expensive, we have observed how the introduction of the ProPCC Programme means a saving of €3,360 per patient during the following 6 months after its inclusion in the programme.

With this early implementation evaluation, we can synthesise several key points of the experience of integrated care at the meso level in our territory, which we interpret in the international framework.

### Increased Primary Care Proactivity

The evidence in adapted care programmes for patients with chronic conditions shows different models that have produced beneficial results, such as favouring maintenance in the community and limiting hospitalizations [14,15]. Our results are in line with several programmes tested in the primary care teams in the North American continent that have shown the importance of the proactivity of intensive primary care monitoring schemes [16]. One of the best-implemented and validated models is the one used by the US Department of Veterans Affairs [17], which has managed to improve results for patients with high health and social needs. Its most solid model is Home-Based Primary Care [18], which they have shown better accessibility, higher quality and lower costs, mainly resulting from the reduction of hospitalizations. The key to our model of interprofessional care teams, regular interprofessional care meetings, and rapid response to crises from primary care are shared with those of this model. Other intensification models such as GRACE [19, 20] or Guided Care [21] have also shown results of efficiency and improvement in the quality of care. The keys to these models also shared with ours, are the protocols of multidisciplinary geriatric interventions with a marked leadership of primary care nursing. It is important to emphasise that our model has been developed and implemented in the context of the public health system of Catalonia, in Southern Europe, where primary care is highly structured, but where hospital care is the one with the most resources. This fact makes it difficult to compare our model to the American schemes or care models from other territories where primary care is not so well structured in a universal public care network.

### Multidisciplinary Case Management Throughout the Community and Hospital Trajectory

One of the common goals of the teams that have deployed the case management model has been the fact of being able to keep elderly complex patients at home, despite being at high risk of suffering disease progression. It has been very important during the implementation that the primary health and social care teams work collaboratively with the community teams for home hospitalisation, home palliative care and home rehabilitation, with the support of hospital services to get the maximum time at home possible. At a European level, we see how several programmes have shown a reduction in hospitalisations and greater community care from integrated interventions based on case management in the community. In Italy, the group of Bernabei et al [22] showed how integrated social and medical care with case management programmes may provide a cost-effective approach to reduce admission to institutions and functional decline in older people living in the community. In Switzerland [23], a mixed horizontal-vertical integrated care experience showed a reduction in unnecessary hospitalizations and a higher probability of dying at home. The British system has a long history in the virtual wards model [24], which shares, with our model, the selection of high-risk patients and the intensification of follow-up in the community based on this risk.

Therefore, for the implementation, we have adopted a CGA-based hospital-at-home intervention developed in Catalonia [25], together with hospice at home and rehabilitation at home to respond to crises. We believe the creation of ecosystems that facilitate the management of complexity at a territorial level is one of the key points to reorder the system and provide care centred on the person in the community. In Valencia (Spain), Tortajada et al [26] also show the experience of the creation of this ecosystem. They have studied the impact of integrated case management intervention at outpatient clinics with nurse case managers from a telemedicine unit. Their results also were an increase in time spent at home and a reduction in the use of hospital resources. This experience shares with ours the role of nursing in case management and the activation of alternative resources to conventional hospitalisation, such as home hospitalisation.

On the other hand, the results of reducing hospital stays are in line with several case management programmes during hospitalisation aimed at high-need high-cost patients, such as several American programs [27, 28]. These experiences, like ours, show how the proactivity of a multidisciplinary team during hospitalisation and the coordination and deployment of subsequent transitional interventions are efficient in facilitating discharges and reducing readmissions.

### Health Care Resources Are Mobilised from the Hospital to Primary Care

The results presented here show the inversion of costs: the 67% increase in spending on primary care and the 61% reduction in spending on hospital care. There are many authors who advocate for the system of the future to be a system based on the primary care teams and the community health and social care network that supports the whole life trajectory of each patient, including the phases of complex care needs and end-of- life. This is why these results can be useful for the planning of future policies, not only in the context of the health plan and chronicity policies of Catalonia [29,30,31], but also in other systems elsewhere [32].

### Reducing Care Costs in Hig-hneed Hig-hcost Populations at End-of-life

It is known from the literature that older populations that are at end-of-life (last months of life) consume high number of resources, and most of these are unplanned hospital-based services, such as emergency department use or hospitalisations [7]. In this sense, our findings on the positive economic impact of our programme on resources use in an urban area in Southern Europe are relevant to adapt this kind of care management models for the care to high-need high-cost populations with chronic advanced conditions in other systems. Despite in the sub-analysis of non-survivors, the positive results in health resources reduction are worse and they do not remain statistically significant, compared to the survivors’, these results enhance the validity and reliability of our study findings because they mitigate the risk of bias related to the fact that patients who are no longer alive does not utilise care resources.

## Limitations and Future Challenges

It should be noted that our programme has been implemented and evaluated with some limitations, which we share below.

### Pragmatic Methodology and Implementation in Pandemic Times

Although the results of this study are promising, the data we have presented are retrospective and in the context of real-life implementation. The approach we have taken in the analysis is pragmatic and the results must be confirmed with prospective studies, comparing the effect of the intervention with a controlled group that does not receive the implemented model. In this sense, future designs should include control groups involving primary care teams in which the programme is not yet implemented. On the other hand, it is necessary to consider the impact that the pandemic has had on the health system: although the implementation activity has been maintained, it must be recognized that analysing integrated care in times of covid-19 can be complex.

### The Challenge of Promoting Horizontal Integration in a Territory with Multiple Actors

One of the main limitations of our project is that it is not born from the Catalan health or social system (Catsalut or departments of Health or Social Affairs), but from one of its main suppliers (Catalan Health Institute). This is why our model has a significant hypertrophy of efforts directed towards vertical integration and has shortcomings in the approximation of horizontal health and social integration. This fact has been taken into consideration when defining the model since in our territory, social services depend on more than 70 different councils. In order to try to overcome the barrier of having to work with several social service intermediates, we have considered the figure of the primary care social worker as it has been shown to be important [33]. This figure acts as an interlocutor with the professionals responsible for the social service’s response of the different municipalities. It is important for us to improve the involvement of municipal social services professionals in the provision of care as one of the formulas that the programme allows is for them to be systematically included in case conferences.

### From Increased Time at Home to the Patient Experience

Although we have used the measure of time spent at home as a proxy variable for what matters to our patients and relatives, in future work we plan to evaluate the experience of the participants in the programme by comparing it with the experience of high-need high-cost patients who live in territories where it has not yet been implemented. On the other hand, it is planned to include the patient experience (PREM) and the outcomes reported by people (PROM) in the continuous monitoring of our care model once the implementation is consolidated.

## Lessons Learned

- The clinical programme has an important impact on everyone involved in integrated care, to work with the common goal of keeping high-need people at home.
- We achieved the shared case management goals of increasing time at home (up to 3%) and reducing time in hospitals (up to 38%).
- Collaboration between teams in the community has succeeded in reducing hospital referrals (up to 37%).
- The proactivity from the clinical programme has increased the number of visits by the primary care teams, which has led to a cost reduction of 46.4% for the public health system due to a reduction in hospital-based resource use.
- These initial data need to be confirmed prospectively, with new controlled studies that use health and social costs and patient experience amongst other person-centred measures.

## Conclusions

The results of this work show that it is possible to increase the time spent at home, reducing the time of hospitalisation, based on increasing the proactivity of the primary care teams and improving their collaboration with different community teams and hospitals, focusing efforts and resources on maintaining older people with high-needs at home. The savings in the use of hospital resources, compensated by the intensification in the use of primary care resources, reduced the average total cost related to health service use to almost half. Future prospective work must confirm these results and analyse what impact the implementation of the programme has on other direct and indirect health and social costs, and on the experience of the participants.
